# Does verbatim sentence recall underestimate the language competence of near-native speakers?

**DOI:** 10.3389/fpsyg.2015.00063

**Published:** 2015-02-04

**Authors:** Judith Schweppe, Sandra Barth, Almut Ketzer-Nöltge, Ralf Rummer

**Affiliations:** ^1^Department of Psychology, University of ErfurtErfurt, Germany; ^2^Project for Successful Teaching and Studies, Kiel UniversityKiel, Germany

**Keywords:** sentence recall, bilingualism, near-native speakers, attention, language competence, working memory

## Abstract

Verbatim sentence recall is widely used to test the language competence of native and non-native speakers since it involves comprehension and production of connected speech. However, we assume that, to maintain surface information, sentence recall relies particularly on attentional resources, which differentially affects native and non-native speakers. Since even in near-natives language processing is less automatized than in native speakers, processing a sentence in a foreign language plus retaining its surface may result in a cognitive overload. We contrasted sentence recall performance of German native speakers with that of highly proficient non-natives. Non-natives recalled the sentences significantly poorer than the natives, but performed equally well on a cloze test. This implies that sentence recall underestimates the language competence of good non-native speakers in mixed groups with native speakers. The findings also suggest that theories of sentence recall need to consider both its linguistic and its attentional aspects.

## Introduction

Verbatim sentence recall (or sentence repetition testing, Diller and Jordan-Diller, 2003) is a task widely used in tests of language proficiency in educational (e.g., Grimm, 2001; Fried, 2008) and clinical contexts (e.g., Meyers et al., 2000) because it discriminates well between good and less good performers (Grimm, 2001). It has been used for measuring second language (L2) competence (Radloff and Hallberg, 1991; Diller and Jordan-Diller, 2003) and is also included in language development tests (e.g., for German: SETK 3-5, Grimm, 2001; SSV, Grimm, 2003; for English: TOLD-P:4, Newcomer and Hammill, 2008; KLST-2, Gauthier and Madison, 1998). An advantage of using sentence recall in language proficiency tests is that it can be conducted with little effort. Furthermore, verbatim sentence recall covers many aspects of language processing: it requires comprehension and production skills, and involves processing at phonological, lexical-semantic, morphosyntactic, syntactic, and propositional levels (e.g., Schweppe, 2006). It is therefore also used in psycholinguistic research for studying syntactic priming (e.g., Potter and Lombardi, 1998; Meijer and Fox Tree, 2003).

In spite of these advantages, we suggest that the verbatim recall of sentences is not that good a measure for estimating differences in language competence between native and highly proficient non-native speakers, for sentence recall systematically underestimates language proficiency of these highly proficient L2 speakers. In online sentence processing, there is usually no need to maintain surface information. However, we assume that when a sentence is processed for verbatim recall, surface representations need to be kept available, a process that is cognitively costly (Aaronson and Scarborough, 1976; Rummer et al., 2013). The need to recall the exact wording of sentences would thus not only require verbal competence but also a substantial amount of general attentional resources. When, in addition to the maintenance, also the processing of the sentence itself is cognitively costly—as is the case in a non-native language, even when language competence is very high (Clahsen and Felser, 2006)—the attentional demands imposed by verbatim sentence recall could be too high and recall performance could thus break down. Consequently, if language competence of (highly) proficient non-native speakers is evaluated based on verbatim sentence recall, it could be underestimated as compared to other tasks and as compared to native speakers whose language processing is more automatized. This should even be the case for non-native speakers with native-like proficiency in other linguistic tasks—so-termed *near-natives*—whose performance on sentence recall would then nevertheless be considerably poorer than that of natives.

In light of these considerations, a specific application of sentence recall appears critical: The task is a core part of an annual language screening for preschool children in Germany in which children with special needs are identified (e.g., “DELFIN 4,” Fried and Briedigkeit, 2007; Fried, 2008). Crucially, the assessment of the test outcomes does not consider whether the children are L1 or L2 speakers. The most prominently discussed outcome of these language screenings was the finding that children with German as an L2 performed dramatically poorer than L1 children. If our assumptions are correct, this finding could partly be due to sentence recall underestimating the language proficiency of good L2 speakers as compared to native speakers. Consequently, one should avoid lumping together native and non-native speakers when using this task.

In the present paper, we test our assumptions by investigating (adult) native and highly proficient non-native speakers of German with respect to their performance in a verbatim sentence recall task. In addition, we test their performance in a complex language task that also addresses comprehension and production skills but does not require explicit maintenance and which should thus not demand substantial attentional resources. However, from the perspective of the *conceptual regeneration hypothesis* (Potter and Lombardi, 1990), one could question the assumption that verbatim sentence recall requires the active maintenance of surface information and that processing a sentence for verbatim recall is therefore attentionally more demanding than processing it for comprehension. We will thus briefly describe Potter and Lombardi's (1990, see also Lombardi and Potter, 1992; Potter and Lombardi, 1998) account as well as our modification (Rummer et al., 2013).

According to the conceptual regeneration hypothesis, sentences are not stored in working memory but are regenerated based on the propositional representations abstracted during comprehension. Sentence recall is therefore simply a combination of sentence comprehension and sentence production. Nonetheless, recall is often verbatim, since lexical-semantic representations of the words in the sentence are activated in the course of comprehension, and participants access these during regeneration with a higher probability. Rummer and colleagues (e.g., Rummer and Engelkamp, 2001, 2003; Rummer et al., 2003; Schweppe and Rummer, 2007; Schweppe et al., 2011) modified Potter and Lombardi's (1990) approach such that presentation of a (to-be-comprehended or to-be-recalled) sentence automatically activates long-term memory representations on all levels of language processing and that these representations can be kept available. Which representations are maintained depends on the affordances of the particular task. Surface representations, such as phonological ones, are kept available only when the task is to recall the exact wording of a sentence (Rummer et al., 2013). The mechanism responsible for prolonging activation of otherwise dispensable surface representations is related to the allocation of attention and is assumed to be cognitively demanding ^1^. This idea is based on more general theories that think of working memory as attentional processes operating on long-term memory (e.g., Cowan, 1999; Barrouillet et al., 2004). Cowan's *embedded processes model* (Cowan, 1995, 1999, 2001) assumes that during encoding a stimulus activates multiple features in long-term memory and that their activation can be maintained by directing attention inwards to representations that are relevant for the current task. Attention is a limited resource that is shared between processing and maintenance of activation. Verbatim sentence recall should thus be attentionally more demanding than sentence comprehension or gist recall, as more representations need to be maintained (Rummer et al., 2013). A reading time study by Aaronson and Scarborough (1976) suggests that this is indeed the case: when reading a sentence for recall, reading times were significantly higher than when participants read the same sentences for comprehension. Another finding that is in line with the idea that sentence recall demands attention comes from a study by Baddeley et al. (2009). They investigated (auditory) sentence recall as a single task and in combination with a visual continuous reaction time task that required general attention and observed poorer recall performance in the dual task condition than in the single task condition.

The idea that attention is shared between processing and maintenance also suggests that the more attention demanding a processing task is, the more it should hinder maintenance (e.g., Cowan, 1999; Barrouillet et al., 2004). One factor that affects the attentional demands of language processing is whether a native or a non-native language is processed: even in non-natives who command their L2 very much like natives and perform native-like in other language tasks, language processing is less automatized than in an L1 (e.g., Bosch et al., 2000; Clahsen and Felser, 2006). For instance, Stowe and Sabourin (2005) report that although the same brain areas are active during sentence comprehension (and other processing tasks) in L1 and L2, the activation when processing an L2 is increased even in highly proficient speakers with age of acquisition around the age of three. Similar findings are reported for Spanish-Catalan bilinguals who were exposed to both languages to the same degree from their third year on (Perani et al., 2003). Importantly, the increase of activation during L2 processing was observed even in the absence of performance differences between L1 and L2 speakers (see also Birdsong, 2006). A meta-analysis of neural processing differences between native and non-native speakers suggests that these can most reliably be found in the left prefrontal cortex, specifically in the left inferior frontal gyrus (e.g., BA 47), which is involved in non-lexical compositional processes such as syntactic processing during sentence comprehension (Indefrey, 2006). The same areas are also involved in executive processes such as attentional control (Miller and Cohen, 2001). Higher activation levels of prefrontal areas in non-native sentence processing may thus either reflect compensation for lower efficiency in these regions (Indefrey, 2006) or “executive control over access to short- or long-term memory representations” (Abutalebi, 2008, p. 472; see also Thompson-Schill et al., 1997; Fletcher et al., 1998) and thus higher attentional load.

If (1) non-native language processing is indeed attentionally more demanding than native language processing even in highly proficient bilinguals, and (2) verbatim sentence recall is a particularly demanding verbal task, the need to recall the exact wording of a sentence in a non-native language may overload the attentional system. This kind of overload is less likely to occur in L1 sentence recall because in this case attentionally demanding maintenance is combined with attentionally less demanding processing. The imperfect automaticity of highly proficient L2 speakers, which does not have consequences for performance in most linguistic tasks, would lead to substantial consequences in an attentionally demanding task such as sentence recall. In other words, verbatim sentence recall should increase performance differences between native and non-native speakers as compared to a task that also taps comprehension and production skills at sentence or discourse level but that does not pose additional maintenance demands.

The present study tests this assumption by comparing native and non-native speakers' performance in sentence recall and on the C-Test, which follows the principles of a cloze-test and consists of short texts with gaps (Raatz and Klein-Braley, 1982). Like sentence repetition, C-test performance relies on both sentence comprehension and sentence production and taps semantic as well as syntactic skills. However, unlike sentence repetition, it does not require the controlled maintenance of surface representations. We therefore predict that when comparing a group of highly proficient non-natives and a group of natives, performance differences will be considerably larger in sentence recall than on the C-test. A particularly strong test of this hypothesis would be to compare natives and non-natives with similar C-test performance. We thus contrasted non-native participants with high scores on the C-Test and a sample of native speakers of German. In addition, we tested participants' performance in sentence recall with both auditory and visual presentation since it was an open question whether one of the two input modalities might cause particular difficulties for the near-native speakers.

## Study

The experiment was based on a 2 × 2 design with the quasi-experimental variable language group (native speakers of German vs. near-native speakers of German) and the within-subjects variable presentation modality (auditory vs. visual). Performance on the C-Test and in verbatim sentence recall served as dependent variables. Furthermore, the C-Test score was included as a covariate in the analyses on sentence recall, and sentence recall performance was included as a covariate in the analyses for the C-Test.

### Method

#### Participants

Seventy eight participants, 54 native, and 24 non-native speakers of German, were tested. The non-native speakers (mean age: 26.67 years, range 18–60) were students or employees at the (German) universities of Erfurt, Hamburg, Leipzig, and Saarbrücken. They all reported to have had at least 5 years of experience with German as a second language (mean number of years of experience = 14.2 years) and had participated successfully in German school or university education or both. Each non-native participant reported to have the German general qualification for university entrance. Their long stay in Germany and their successful educational history indicate that the subjects mastered their L2 to an extremely high degree. The non-native group was heterogeneous with respect to their first language (e.g., Indonesian, Finnish, Serbo-Croatian). A broader sample of native participants (with respect to age and educational background) was tested ^2^. The pool of the native speakers (mean age: 19.09 years, range 13–27) included 20 students at the University of Erfurt (18–25 years of age), 15 high school students attending a school of the college-bound track of the German school system (“Gymnasium”) in Erfurt (at the transition from grade 9–10; 13–14 years of age) as well as 19 vocational school students (18–27 years of age), 12 of whom were also studying for a qualification for university entrance.

#### Materials and procedure

Participants had to conduct two tests: first, a paper-pencil based C-Test, and second, a computer-based verbatim sentence recall task.

***C-Test***. The C-Test was first developed by Raatz and Klein-Braley (1982) and has since been studied in many languages (for an overview see the C-Test bibliography by Grotjahn, 2007). Native speakers' C-Test scores correlate with school grades in native language classes up to grade eight (Wockenfuß and Raatz, 2006). For L2 learners, C-Test scores correlate highly with performance in second language courses (Eckes, 2010) and are widely used to assign learners to course levels. Furthermore, C-Test scores correlate highly with scores on institutionalized L2 proficiency test batteries, for instance with the TOEFL (*r* = 0.55–0.91), TOEIC (*r* = 0.62), and the Michigan Test (*r* = 0.54–0.61), and with the Oxford Placement Test (*r* = 0.83) in English and the TestDaF (*r* = 0.76) in German (for an overview see Eckes and Grotjahn, 2006). For the purpose of our study it may be problematic that older adolescent and adult native speakers should score at ceiling on most C-Tests. However, it is possible to construct C-Tests so that even the native speakers do not score perfectly (Baur and Meder, 1994). The C-Test used in our study consisted of four short texts with 99 gaps (for the original texts and an English translation see the Appendix in Supplementary Material). The texts differed in their theme and style, their difficulty increased. In line with the standard C-Test procedure, 5 min were given for each short text. In general, participants took less than 5 min to complete each of the texts. We chose the texts to be sufficiently difficult to induce errors by the native speakers. Only one of the 54 natives (and one of the 24 non-natives) achieved a perfect score, which demonstrates that this was indeed the case in our sample.

***Sentence recall***. After completing the C-Test, subjects received instructions for the computer-based part of the study. Instructions were presented on a computer screen and subjects were encouraged to ask questions when the procedure was not clear to them. The sentence recall task consisted of 40 sentences, which were adapted from the German version of Daneman and Carpenter's (1980) reading span test (Hacker et al., 2002). Since native speakers are able to recall sentences of up to 16 words or more (Brener, 1940), the sentences used here were slightly modified in length to induce errors in L1 sentence recall. Each modified sentence included 16–20 words (mean length 17.8; *SD* = 1.12). The materials (including the audio files) are available for download at the IRIS digital repository (http://www.iris-database.org/iris/app/home/detail?id=york%3a815588 and). Sentences were divided into four blocks with mean sentence length of 17.9, 17.7, 18, and 17.5 using a latin square technique. The sentences were recorded for auditory presentation by a female native speaker. In the visual condition, they were presented center screen white on black with font Arial and size 22 pt. Sound files lasted between five and eight seconds and presentation times of the written sentences matched presentation times of the auditory versions. The four blocks of sentences were balanced across conditions, each participant was presented two blocks, and no participant was presented the same sentence more than once. A *post-hoc* analysis revealed no differences in the ease of recall between the four blocks. In the first block, sentences were presented auditorily via headphones. The second block was presented visually. This order was the same for all participants. Within blocks, the order of the sentences was randomized. After each sentence was presented, the participants had time as needed to write down the exact wording, and then they started the next trial by pressing the space bar. Each block began with two practice trials. Finally, participants completed a questionnaire regarding their language history, were debriefed and received payment or course credit for their participation.

### Results

First, we will present analyses with sentence recall performance as the dependent variable, which will be followed by the analyses with C-Test performance as dependent variable. One native speaker (C-Test score 99; (auditory) recall performance 97.28%) and one non-native speaker (C-Test score 97; (auditory) recall performance 82.32%) only completed the auditory items. To base the analyses for both dependent variables on the same pool of participants, we also excluded these two participants from the analyses on C-Test scores.

#### Sentence recall

We scored participants' responses according to a strict criterion but disregarding word order. Words were scored as correct only when they occurred in the same grammatical form as in the original sentence, while purely orthographic mistakes were ignored. We subjected the proportion of correctly recalled words per sentence to an ANCOVA with “language” as between-subjects variable, “modality” as within-subject variable as well as age and C-Test score as covariates.

As expected, there was a significant main effect for language in that the L1 speakers (79.52% correct, *SE* = 1.52) outperformed the L2 speakers [69.9%, *SE* = 2.48; *F*_(1, 72)_ = 9.61, *p* = 0.003, η^2^_p_ = 0.12]. There was also a significant effect for the covariate C-Test score [*F*_(1, 72)_ = 34.41, *p* < 0.001, η^2^_p_ = 0.32], while age did not significantly influence recall performance (*F* < 1).

We varied the input modality in the sentence recall task in order to explore whether one of the two modalities caused particular difficulties for the near-native speakers. As there was no interaction between language and modality (*F* < 1) and both groups performed better with auditory than with visual presentation (77.29% correct, *SE* = 1.39, vs. 72.12% correct, *SE* = 1.52; *F*_(1, 72)_ = 5.65, *p* = 0.02, η^2^_p_ = 0.07), this does not seem to be the case.

#### C-test

For the C-Test, we awarded one point for each correctly filled gap such that the maximum score was 99. We subjected the resulting C-Test scores to an ANCOVA with “language” as between-subjects variable, and age and sentence recall performance (proportion of correctly reproduced words) as covariates. There was a significant effect for the covariate sentence recall performance [*F*_(1, 72)_ = 34.36; *p* < 0.001, η^2^_p_ = 0.32], whereas the covariate age only approached significance [*F*_(1, 72)_ = 3.34; *p* = 0.07, η^2^_p_ = 0.04]. Crucially, the main effect for language did not reach significance (*F* < 1). L2 speakers scored as high on the C-Test (91.09, *SE* = 0.93) as did the L1 speakers (90.43, *SE* = 0.56).

## Discussion

The experiment aimed at demonstrating that sentence recall underestimates the language proficiency of very good L2 speakers as compared to a similarly complex verbal task that does not pose additional attentional demands. As predicted, near-natives with a highly successful educational history, long stay in Germany, and hardly any foreign accent in German performed much lower than native speakers with comparable C-Test scores when instructed to repeat the exact wording of sentences in their L2. Even though the C-Test was included as a covariate, the fact whether a participant was a native or a non-native speaker affected sentence recall performance. In contrast, only sentence recall performance accounted for variance in the C-Test scores.

One explanation for this finding is that sentence recall is a more fine-grained measure of language proficiency than the C-Test. In this case, sentence recall would uncover subtle deficits the C-test did not detect. A finding that can be interpreted as supporting this idea is the overall greater error rate in sentence recall as compared to C-test performance. Nonetheless, there was a considerable range for the C-Test scores both within the group of native speakers (75–99) and within the non-natives (81–99).

A potentially critical aspect concerns the use of written output in sentence recall, which might be more problematic for non-native than for native speakers. This output modality was chosen to maximize structural similarity to the C-Test. To ensure that the results reported here are not restricted to written output, we replicated the study with another small group of natives (*N* = 9) and near-natives (*N* = 9) using oral sentence recall. This study revealed similar results. Given these additional data and the fact that both tasks, sentence recall and the C-Test, required written output, it seems implausible that the relatively poor sentence recall performance for otherwise highly-proficient L2 speakers can be attributed to problems with the output modality. Furthermore, it has been suggested that written output reduces the demands for output control and thus the cognitive demands imposed by a recall task since it provides a written record of the already recalled words (Marsh et al., 2011). These considerations even suggest favoring written over oral recall when comparing natives' and near-natives' sentence recall performance.

A further caveat could be the fact that the group of native speakers was more heterogeneous with respect to their age and educational background than the non-native speakers were. In addition to a sample of university students, we included high school students and vocational school students. However, analogous analyses with a smaller sample in which only the L1 university students were included revealed similar results. The only difference was a descriptive advantage for L1 over L2 speakers on the C-Test, but with recall performance as a covariate [*F*_(1, 39)_ = 34.75; *p* < 0.001], the main effect for language group did again not reach significance (*F* < 1). The difference in age and educational background thus influenced performance in the native sample but could not have caused the basic data pattern.

Assuming that the large discrepancy between sentence recall and C-Test performance is indeed caused by the need to process an L2, we now return to our underlying hypotheses. According to Potter and Lombardi (1990, 1998), sentence recall is a reconstruction based on the propositional structure generated during comprehension, plus lexical and syntactic priming. We assumed that under the instruction to recall the exact wording of a sentence, additionally, surface representations such as phonological ones would be maintained. This process of maintenance requires attention (Barrouillet et al., 2004). Sentence recall performance should thus decline if another process simultaneously draws on attention, as does second language comprehension and production. The fact that even highly proficient L2 speakers show poorer sentence recall performance than L1 speakers with the same mean C-Test scores supports this assumption. The present findings therefore highlight the fact that sentence recall is a task that requires substantial language skills as well as attention. Therefore, an explanation of how sentence recall works needs to consider both its linguistic and its working memory/attentional aspects.

As outlined above, it is plausible that the poor sentence recall performance of near-natives is due to a lack of automaticity of L2 (compared to L1) processing. The lesser degree of automaticity influences performance when the verbal task requires additional attentional control, as is the case with sentence repetition. In near-natives this difference in automaticity is not crucial for regular language processing and for successful education, as is indicated by the fact that the non-native speakers we tested performed native-like on the C-Test and had successful educational careers in their L2 German (all had acquired the German general qualification for university entrance). However, it remains indisputable that the degree to which language processing is automatized is also a criterion for language proficiency. It may be that—at the high end of L2 proficiency—the discrepancy between C-Test performance and sentence recall performance is a feasible way of measuring automaticity. Furthermore, the C-Test cannot fully capture the demands of online processing of transient information, as the to-be-completed texts are available for re-inspection throughout the task. Even though it is an established instrument for measuring language proficiency that correlates with more extensive test batteries (for an overview see Eckes, 2010), it is not a perfect measure of general language competence. There are certain verbal tasks that may require a similar degree of attentional control or pose similar maintenance demands as sentence recall does. Possible candidates are dual task situations (such as talking while driving, e.g., Becic et al., 2010), the resolution of long-distance anaphora (e.g., Daneman and Carpenter, 1980) or the processing of long-distance dependencies (e.g., Deane, 1991; Hawkins, 2004). With high attentional load in these tasks, sentence recall might predict L2 speakers' performance at least as well as the C-Test, even in mixed groups of natives and non-natives^3^. Further research that uses a larger battery of verbal tasks is required to address these questions.

To have a strong test of our prediction that sentence recall underestimates L2 proficiency, we chose highly proficient L2 speakers. However, the same should hold true for a broader group of non-native speakers. As with increasing L2 proficiency automaticity goes up, sentence recall should also pose problems at lower levels of L2 proficiency. The implications of our findings are hence not restricted to the constrained group of near-natives. Still, a plausible exception to this exists: for speakers on the lower end of L2 proficiency, sentence recall could actually overestimate language skills. Since one can repeat lists of non-words, it is possible to base “sentence” recall on rote repetition and thus to repeat (parts) of sentences that one is not able to understand.

To conclude, our data suggest that sentence recall is not as good a measure as previously assumed when it is used as a predictor for how well a non-native speaker is able to communicate or to participate in education. This is particularly problematic when non-native and native speakers are analyzed jointly. When the goal is to identify differences within a group of non-natives or within a group of natives, sentence recall can still be an adequate measure. When sentence recall is used in language screenings for mixed groups (e.g., Fried and Briedigkeit, 2007), the problem might be addressed by gathering separate norms for native and non-native speakers.

### Conflict of interest statement

The authors declare that the research was conducted in the absence of any commercial or financial relationships that could be construed as a potential conflict of interest.

## Footnotes

^1^There is a discussion going on whether forgetting in working memory is due to a decay of activation that can be prevented via rehearsal (e.g., Barrouillet and Camos, 2009) or whether it is due to interference between competing representations (e.g., Lewandowsky et al., 2009). We talk about the maintenance of activation in this context and refer to a working memory model that emphasizes the role of decay. However, the basic assumptions apply irrespective of whether one assumes that it is attentionally demanding to prevent activation from decay and thus to keep the activated representations available for verbatim recall or that it is attentionally demanding to reduce interference from competing representations and thus to prevent intrusions from lexical competitors or syntactic alternatives during verbatim sentence recall.

^2^This was originally done to increase the probability of identifying native speakers who could be matched with respect to their sentence recall performance.

^3^We thank one of the reviewers for this suggestion.
