# Supplementary material for: Does verbatim sentence recall underestimate the language competence of near-native speakers?
Source: Front Psychol. 2015 Feb 4;6:63. doi: 10.3389/fpsyg.2015.00063 (PMC4316704; doi:10.3389/fpsyg.2015.00063)
Supplement: Supplementary file 1 [file DataSheet1.DOCX]

1. **Appendix**
   1. **C-Test texts: German original**

Text 1

Nach dem Kindergarten gehen alle Kinder im Alter von sechs oder sieben Jahren in die Grundschule. Dort ble_____ sie vi___ Jahre. Da___ gehen ca. 25 Pro_____ auf d___ Hauptschule, ca. 45 Pro____ auf d___ Realschule u___ ca. 30 Pro_____ auf d___ Gymnasium. I__ vielen Bundesl_______ gibt e__ auch Gesamtsch_____. Nach d___ Hauptschule ka___ man arbe_____ und Ge___ verdienen od___ eine Le____ machen. Na___ dem Abi____ am Gymn______ kann m___ einen Be____ lernen oder an der Universität studieren.

Text 2

In sechs Stunden kann man per Straßenbahn quer durch das Ruhrgebiet fahren. 5,6 Millionen Men______ wohnen und arbe_____ hier. D___ Städte si___ durch e___ kompliziertes Ne___ von Straßenba_____ und Bus-Li_____ verbunden. D___ längste Straßenbahn-Li____ der We___ ist 115 km la___. Man ka___ sie v___ Düsseldorf b___ nach Dortmund befa_____. Kommen S___ mit, ste_____ Sie e___; vergessen S___ das Umst______ an d___ richtigen Halteste____ nicht! Uns____ Fahrt beginnt in Düsseldorf.

Text 3

Zeitungsvolltexte via Computer sollen Blinden den Zugang zur täglichen Nachrichtenwelt verschaffen. Auf Initi______ des Deut______ Blindenverbandes u___ der Stif_____ Blindenanstalt wi___ ab die____ Samstag i__ München i__ Rahmen d___ „Reha“ d___ Demonstrationsversion ei____ „Elektronischen Tageszei_____ für Bli____“ gezeigt. D___ Inhalt ei____ überregionalen Tageszei_____ wird a___ einem Fernsehka____ übertragen u___ ka___ von ausgesu______ Testpersonen üb___ ein blindenger_______ Ausgabegerät a_ Computer gele____ werden.

Text 4

Der Streik der Transportunternehmen in Italien, der eine fast einwöchige Versorgungskrise vor allem in der Produktion, bei Nahrungsmitteln und Benzin auslöste, ist am Sonntag vorerst beendet worden. Regierung u___ Vertreter d___ rund 210.000 Transportuntern______ werden i__ den komm______ Wochen ern____ über d___ umstrittene Strukturref____ des Güterverk_____ und Augleichszahl______ wegen erhöh_____ Kraftstoffpreise verhan_____. Bei ein___ Scheitern wol____ die Transportuntern______ erneut stre_____. Italiens Versor_____ ist weitgeh____ von LKW abhä_____, die ru___ 80 Prozent al____ Waren i__ Land transpo________. Die Regi______ will ei____ großen Teil der Warenströme mittels einer Steuerreform auf die Schiene verlagern.

## C-Test texts: English translation (without gaps)

Text 1

After kindergarten, all children aged six to seven go to elementary school. They stay there for four years. Afterwards, about 25 percent go to a Hauptschule, about 45 percent to a Realschule and about 30 percent to a Gymnasium. In many states, there are also comprehensive schools (Gesamtschulen). After the Hauptschule, one can work and earn money or do an apprenticeship. After the Abitur (final exams) at the gymnasium, one can learn a profession or study at the university.

Text 2

It takes six hours to ride straight through the Ruhr district by tram. 5.6 million people live and work there. The cities are connected by a complicated net of trams and bus lines. The longest tram line in the world is 115 km long. One can ride it from Düsseldorf to Dortmund. Come with us, get on board; don't forget to change at the correct station. Our trip starts in Düsseldorf.

Text 3

Full newspaper articles on the computer should give blind people access to the daily news. Following an initiative by the German Society for Blind People and the Foundation “Blindenanstalt” (Establishment for Blind People), the demo version of an electronic daily newspaper for the blind will be presented from this Saturday onwards in Munich as part of the “Rehab”. The content of a nationwide daily newspaper will be broadcast on a TV channel and can then be read by a few selected participants by making use of a blind-friendly output device at the computer.

Text 4

The strike of the transport companies in Italy, which caused an almost week-long supply crisis especially when it comes to production, food and gasoline supplies, was ended, for the time being, on Sunday. In the upcoming weeks, the government and representatives of the circa 210 000 transport companies will negotiate about the controversial freight traffic structural reform and compensation payments due to higher fuel costs. Should the negotiations fail, the transport companies plan further strikes. Italy's supplies are largely depended on trucks, which transport about 80% of all goods in the country. By means of a tax reform, the government is looking to shift of lot of the supply flow to the tracks.
